# Supplementary material for: Mesenchymal Stem Cell Sheet Centrifuge-Assisted Layering Augments Pro-Regenerative Cytokine Production
Source: Cells. 2022 Sep 12;11(18):2840. doi: 10.3390/cells11182840 (PMC9497223; doi:10.3390/cells11182840)
Supplement: Supplementary file 1 [file cells-11-02840-s001.zip › cells-1868213-supplementary.pdf]

Supplemental Materials and Methods

Cell sheet centrifugation without tilting

Cell sheets detached from TRCD by temperature reduction were transferred to 16-hour FBS-coated insert membranes on TCP, or 16-hour FBS coated TCP dishes. These cell sheets on TCP were not tilted to remove excess interfacial medium. Rather, TCPs containing cell sheets were placed into a polydimethylsiloxane (PDMS) mold designed to secure the TCP in the center of a 6-well plate lid [38-40], and this lid was loaded, with balance, into a swing-type plate centrifuge with 16 cm rotor radius (Eppendorf) set to 37°C internal temperature. The 1-layer cell sheets were centrifuged at 114 x g (g-force, or relative centrifugal force) for 60 seconds. Cell sheet condition was observed macroscopically to determine the level of stability and adhesion on the culture surface (n=3 sheets for each condition).

Mechanical rotation test

Adherence between a MSC sheet and the insert membrane culture surface following centrifugation was quantified as the rate of attachment following rotational agitation in medium. First, cell sheets detached from TRCD by temperature reduction were transferred to 16-hour FBS-coated insert membranes on TCPs and tilted for 5 minutes. Cell sheets were then centrifuged at 29, 114, 458, 1030, or 1832 x g using a swing type plate centrifuge (Eppendorf) set to 37°C internal temperature. Immediately afterward, 2 mL warmed growth medium was added to the dish containing the cell sheet and set on a horizontal orbital shaker (VEVOR, Shanghai, China) for agitation at 90 RPM for 10 minutes. To determine adherence, the cell sheet was observed for partial or whole detachment from the insert membrane culture surface (n=3 sheets for each condition).

Supplemental Results

Cell sheet deformation under centrifugation due to excess interfacial media

Cell sheet centrifugation without sliding or deformation requires a stable interaction between the basal plane of the sheet and the culture surface. Cell sheets were manipulated onto the culture surface in aqueous conditions, interstitially trapping residual medium that buffers direct contact. Upon centrifugation at 114 x g for 60 seconds, cell sheets experienced severe sliding in the direction of centrifugal g-force on insert membrane surfaces and TCP surfaces (Figure S1). Cell sheet tilting methods were implemented to remove excess media and enable stable sheet centrifugation without deformation (Figure 2).

Quantification of cell sheet adherence to the culture surface

Cell sheet adherence to the culture surface is necessary for further culture and analysis of 1-layer centrifuged sheets, as well as for consistent and stable subsequent sheet layering. Adherence under a range of centrifugation speeds and durations was evaluated quantitatively by a mechanical rotation test. Cell sheets centrifuged for 120 seconds at 29 x g maintained adherence to the insert membrane surface in all three trials; however, cell sheets centrifuged at 29 x g for 60 seconds detached partially from the surface in one of three trials, and when centrifugation duration was decreased to 30, 15, and 5 seconds, cell sheets detached partially from the surface under mechanical rotation testing in all three trials, indicating weak adhesion (Table 1, Figure S2). Centrifugation at 114 x g for 120 seconds adhered cell sheets in all 3 trials but demonstrated weaker adhesion with only 60 seconds (66% adherence) or 30 seconds (33% adherence) of centrifugation, and consistently failed to adhere the cell sheet with only 15 and 5 seconds of centrifugation (Table 1, Figure S2). Increasing centrifugation speed to 458 x g consistently maintained cell sheet adherence to the surface in 60 seconds and in 30 seconds, although partial cell sheet detachment was observed with only 15 seconds (66% adherence) or 5 seconds (33% adherence) of centrifugation (Table 1, Figure S2). Cell sheets centrifuged at 1030 x g for 30, 15, or 5 seconds were adhered in all three trials following mechanical rotation testing, indicating that this speed forced tight adherence between the cell sheet and the culture surface (Table 1, Figure S2). Cell sheets centrifuged at 1832 x g for 5 seconds displayed visible deformation due to sliding in all three trials, therefore considered a failure for stable cell sheet centrifugation (Table 1, Figure S2). Compared to the conventional incubation method that requires 1 hour to form tight adherence

between the cell sheet and the culture surface, centrifugation shortened adherence time to 120 seconds at a maximum and 5 seconds at a minimum.

Supplemental Figure Caption

Figure S1. Cell sheet centrifugation without tilting to remove excess interfacial culture medium. Single layer cell sheets that did not receive any tilting underwent 114 x g centrifugation for 60 seconds on (a) an insert well membrane and (b) on TCP. In both cases, cell sheets experienced sliding deformation due to poor surface adhesion. Scale = 35 mm well and dish.

Figure S2. Visual representation of single layer cell sheet attachment rate following medium addition and mechanical roration test, summarized in Table 1. Cell sheets centrifuged at 29, 114, 458, 1030, or 1832 x g for 5, 15, 30 60, and 120 seconds. Images show the state of the cell sheet, either partially detached or fully adhered, at the end of the 10-minute mechanical rotation test in medium. Images are representative of triplicate results. Scale bars = 1.0 cm

Supplemental Figures

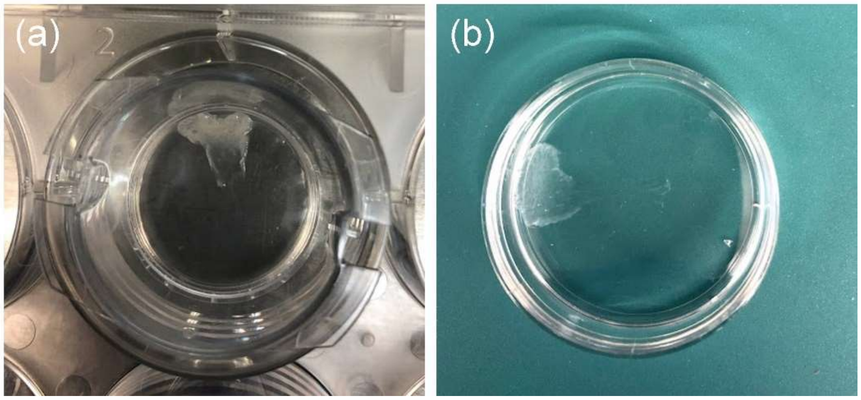

Figure S1. Cell sheet centrifugation without tilting to remove excess interfacial culture medium.

Time / x g	5 seconds	15 seconds	30 seconds	60 seconds	120 seconds
29	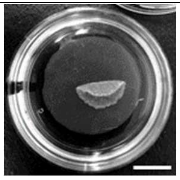	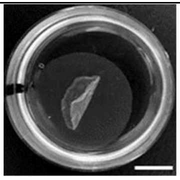	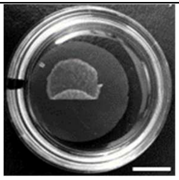	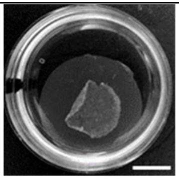	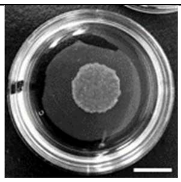
114	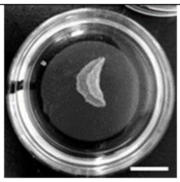	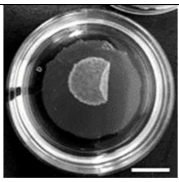	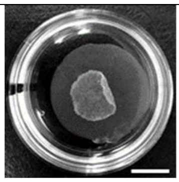	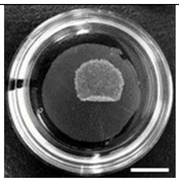	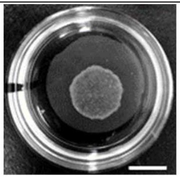
458	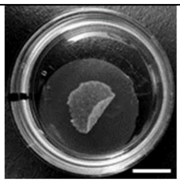	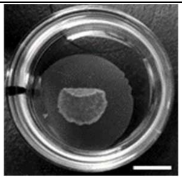	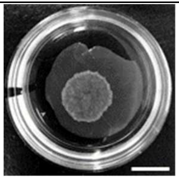	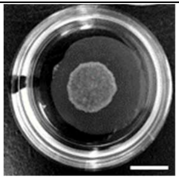	--

1030	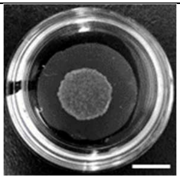	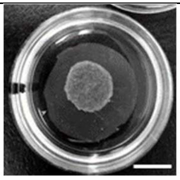	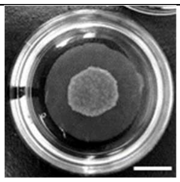	--	--
1832	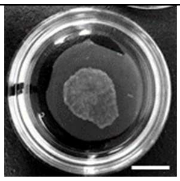	--	--	--	--

Figure S2. Visual representation of single layer cell sheet attachment rate following medium addition and mechanical rotation test, summarized in Table 1. Note that the cell sheets are on insert membrane.
